# Polymyositis in a child with thalassemia after hematopoietic stem cell transplantation: A case report

**DOI:** 10.1097/MD.0000000000027388

**Published:** 2021-10-15

**Authors:** Yonghong Tan, Jinzong Lin, Xiuli Hong, Jingyuan Lu, Quanyi Lu

**Affiliations:** Department of Hematology, Zhongshan Hospital of Xiamen University, Xiamen, Fujian, China.

**Keywords:** chronic graft-versus-host disease, hematopoietic stem cell transplantation, polymyositis

## Abstract

**Rationale:**

Polymyositis (PM) is a rare neuromuscular phenotype of chronic graft-versus-host disease (cGVHD). Although glucocorticoids have been shown to be effective in the treatment of PM, most people experience poor treatment response and poor prognosis.

**Patient concerns:**

A six-year-old boy with thalassemia received allogeneic hematopoietic stem cell transplantation (HSCT) and consequently developed sudden myasthenia of limbs 17 months after the transplant.

**Diagnoses:**

Medical history, current symptoms, laboratory examinations, and imaging findings of the patient indicated cGVHD complicated with PM.

**Interventions:**

He was then given high-dose corticosteroid therapy, including tacrolimus, ruxolitinib, and rituximab.

**Outcomes:**

Twenty-three months after transplantation, creatine kinase levels returned to normal range, and the MRI showed that the original muscle edema signal was significantly improved. The patient's muscle weakness continued to improve, and his overall condition was good.

**Lessons:**

This report suggests that glucocorticoids combined with immunosuppressants may be effective against polymyositis. Rituximab and ruxolitinib may be a good choice in treating polymyositis.

## 1 Introduction

Currently, hematopoietic stem cell transplantation (HSCT) is the only method available to cure hereditary blood disorders in children. Although the long-term survival rate can reach 90% after the transplantation, about 10%–30% of the children also had chronic graft-versus-host disease (cGVHD) of various phenotypes, which can severely influence the quality of their lives and, in some severe cases, cause death.^[1,2]^ cGVHD can affect the skin, oral cavity, eyes, gastrointestinal tract, liver, lung, joints, fascia, genital tract, and muscles.^[3–5]^

Polymyositis (PM) is a rare neuromuscular phenotype of cGVHD. It mainly manifests as symmetrical proximal weakness of the muscles with or without myalgia, elevated serum creatine kinase (CK), or myogenic damage (visible under electromyography). Other cGVHD manifestations generally accompany PM, and some patients cannot achieve full recovery after treatment. The prognosis of PM is associated with the severity of cGVHD.^[6–8]^

Herein, we report a single case of cGVHD in a child with thalassemia treated with allogeneic HSCT. cGVHD appeared as symmetrical muscle weakness in the limbs without evident myalgia. Clinical remission was achieved after treatment with corticosteroids, rituximab, and ruxolitinib.

## 2 Case report

A six-year-old boy was diagnosed with β-thalassemia after birth and was found to have double heterozygous mutations of CD28 and CD17. The boy was treated with unrelated allogeneic HSCT on November 16, 2018. The donor and recipient were of the same blood type and sex. Pre-transplantation examinations were negative for EB virus and cytomegalovirus (CMV) DNA, and the degree of risk of thalassemia was grade 2.^[9]^ The FBuCy-based conditioning regimen and routine methods for the prevention and treatment of GVHD were adopted.^[10,11]^ In total, 10 × 10^6^/kg CD34+ cells and 2 × 10^7^ umbilical cord-derived mesenchymal stem cells were infused. The neutrophil count was >0.5 × 10^9^/L on day 13, platelet count was >20 × 10^9^/L on day 19, and two continuous STR showed that >99% of cells were donor derived, which indicated that the transplantation had been successful. The child was discharged on day 30, and oral ciclosporin and mycophenolate mofetil were prescribed to prevent GVHD. The patient's disease condition remained stable.

On day 103, the child developed a skin rash all over his body accompanied by pruritus and hyperemia of the trunk and bilateral palms. Liver function examination showed that the alanine aminotransferase (ALT) level was 478.6 U/L; the aspartate aminotransferase (AST) level was 576.7 U/L; and the γ-glutamyltransferase (γ-GGT) level was 480.9 U/L. These manifestations met the diagnostic criteria of cGVHD (III skin). Consequently, the child was treated with 1 mg/kg/d methylprednisolone and 10 mg/M^2^ methotrexate for 4 continuous weeks (once per week). Tacrolimus (0.3 mg/kg/d) was also used to replace ciclosporin for combined treatment. After treatment, the skin rash disappeared, and indicators of liver function returned to normal. Oral administration of tacrolimus and low-dose corticosteroids were applied for maintenance treatment.

The patient was re-examined on day 150. The liver function indicators were still abnormal (ALT 265.9 U/L, AST 137.3 U/L, and GGT 551.2 U/L), and the child developed multiple severe oral ulcers. He was then prescribed oral ruxolitinib (5 mg/d) for 1 month, after which liver function and oral ulcers all substantially improved. Therefore, drug therapy was discontinued, and no maintenance treatment was prescribed.

**Figure 1:**
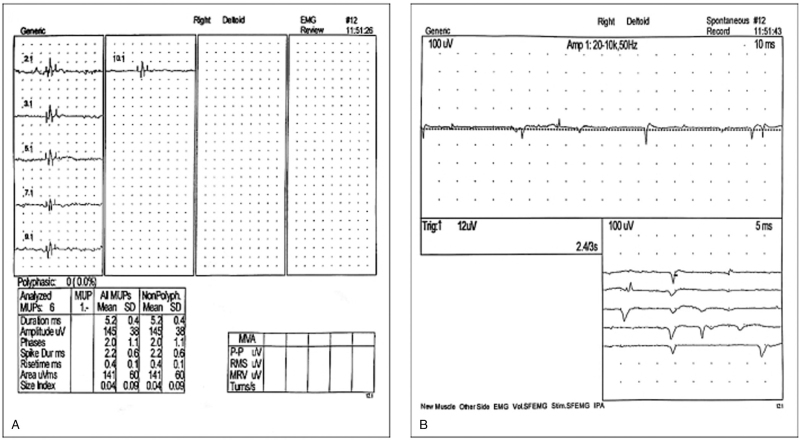
Electromyography. Myogenic damage and spontaneous activity of the right deltoids (A & B).

The child developed sudden myasthenia of limbs in April 2020 17 months after the transplant. He was not able to raise either upper limb, and the bilateral lower limbs were weak. In addition, he had difficulty walking. No fever or myalgia was found. Examination after admission showed multiple severe ulcers of the lower lip, tongue, and buccal mucosa. Evident movement disorder of the proximal limbs was also observed. The muscle strength was grade 4 at the proximal limbs and grade 5 at the distal limbs. The muscular tension of the limbs was normal, and reflex response was active in both knees, and no paresthesia or neuropathological signs were seen. Cranial MRI showed no abnormalities. Peripheral blood examination showed WBC 6.35 × 10^9^/L, Hb 138 g/L, and platelets 179 × 10^9^/L. Liver function examination showed total bilirubin (TBIL) 9.5 μmol/l, ALT 306.1 U/L, AST 365.9 U/L, and GGT 331.6 U/L. Biochemical examination showed lactic dehydrogenase (LDH) 1074.5 U/L, CK 4570.3 U/L, creatine kinase isoenzyme-MB (CK-MB) 248.4 U/L, and troponin T (TnT) 749.6 ng/L. Antinuclear antibodies (ANA) were positive, and the titer against major karyotype was 1:320. The ANA antigen spectrum analysis showed a positive anti-SSA-Ro52 antibody. Electromyography (EMG) showed myogenic damages in the right deltoid muscle and right quadriceps femoris muscle (Fig. 1). According to these examination results, the child was diagnosed with PM. Consequently, intravenous dripping of methylprednisolone (2 mg/kg/d) and oral intake of ruxolitinib (5 mg/d) were performed in combination with mycophenolate mofetil (500 mg/d). The oral and lip ulcers substantially improved 1 week later, and the muscle strength in the limbs slightly improved. Indicators of liver function, LDH, CK, and TnT all substantially decreased.

**Figure 2:**
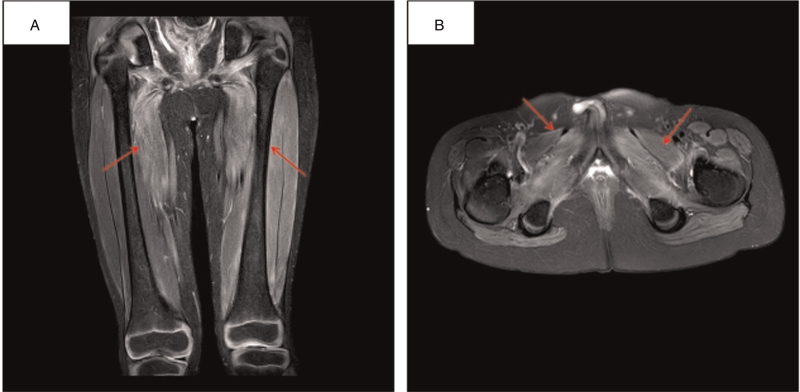
MRI imaging. Fat-suppressed T2-weighed imaging showed high intensity in the left thigh and pelvic muscles (a, b).

**Figure 3:**
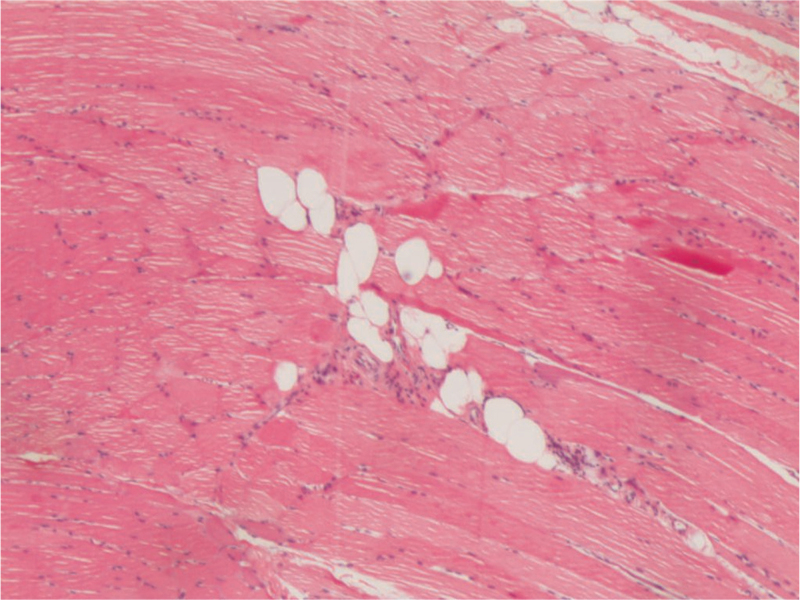
Pathological findings. Slight atrophy of myofibers around the fasciculus, increased number of myofibers in some muscle fibers (myonuclear displacement), and a small number of lymphocytes infiltration.

Re-examination was performed again 2 weeks later, which showed TnT 693.5 ng/L, ALT 383.5 U/L, AST 284.5 U/L, GGT 589.5 U/L, LDH 963.9 U/L, CK 5150.5 U/L, and CK-MB 320.9 U/L. Further examination of the myositis-associated antibody spectrum was also performed, showing negative results for 26 antibodies, including anti-Jo-1 and anti-scl-75 antibodies. Plain MRI scanning of bilateral lower limbs showed slightly high signal intensity at the left thigh and pelvic muscles on fat-suppressed T2-weighed image, which suggested muscle edema (Fig. 2) and changes in myositis. Biopsy of the quadriceps femoris showed slight atrophy of muscle fibers around the muscle bundles and increased number of myonuclei in some muscle fibers (internal translocation of myonuclei) (Fig. 3). These results suggested incomplete remission. Tacrolimus and ruxolitinib were then continued. Also, the patient received 100 mg rituximab once per week and tacrolimus 0.3 mg/kg.d; there were two rounds of treatment.

After 2 weeks, the patient's CK level had fallen significantly to 473.7 U/L and muscle strength improved. The child was then discharged. Oral intake of tacrolimus and ruxolitinib was continued after discharge for maintenance treatment. Re-examination was performed during month 21, which showed ALT 186.4 U/L (increased), AST 106.1 U/L (increased), GGT 627.9 U/L (increased), LDH 439.1 U/L (increased), CK 70.3 U/L, CK-MB 25.9 U/L, and TnT 80.3 ng/L. The CK level was generally normal. Muscle strength was grade 5 in the proximal limbs and generally normal at the distal limbs. The child was able to walk normally: he could engage in daily activities normally.

**Figure 4:**
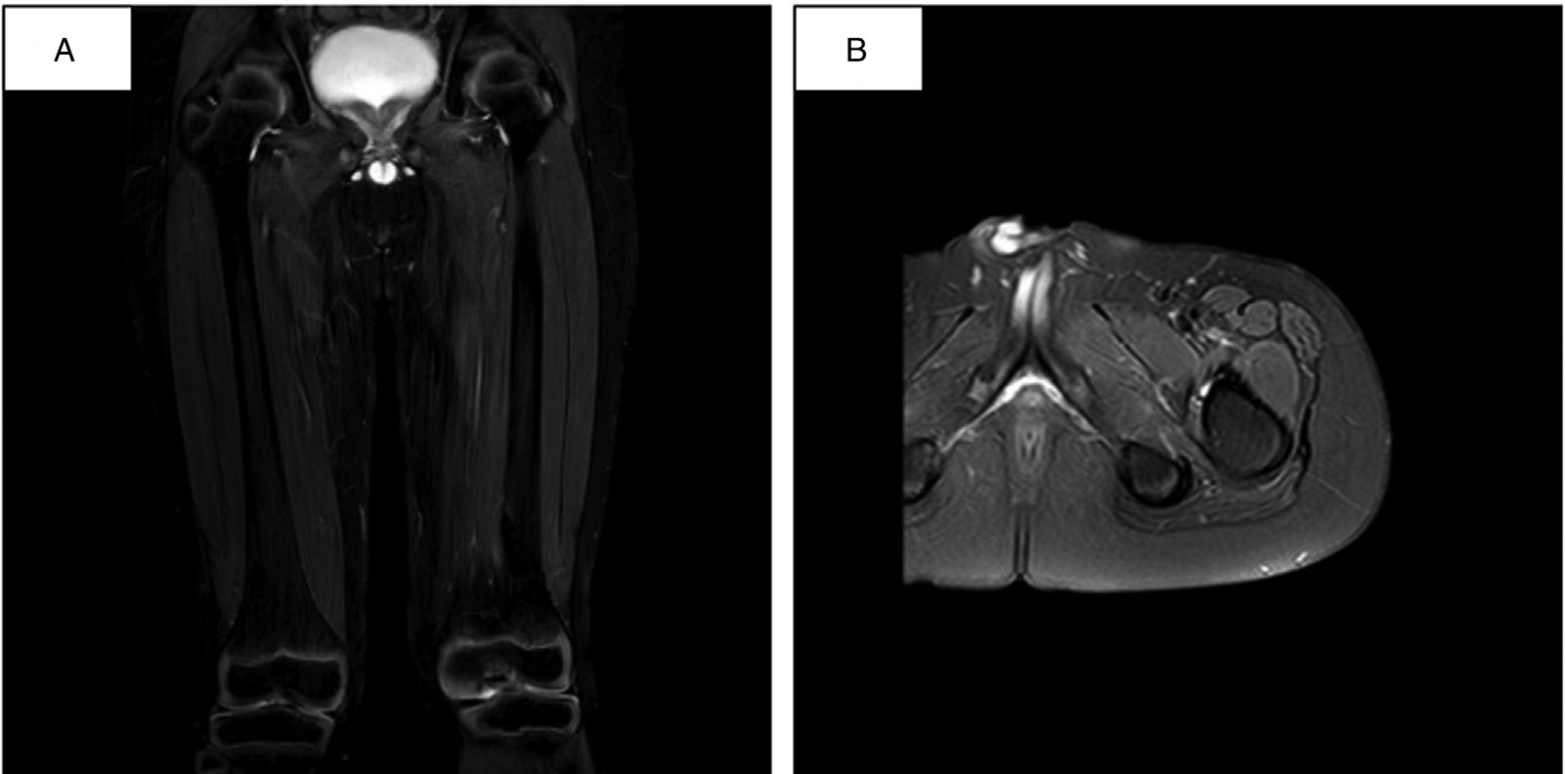
MRI imaging. Fat-suppressed T2-weighed imaging showed the high signals of the original left thigh muscle group and the pelvic muscles basically disappeared, and the signal of muscle edema was significantly improved (a, b).

The child returned to the hospital in October 2020 for re-examination (month 23), which showed normal serum CK. MRI showed that the signals of muscle edema had improved visibly (Fig. 4), the conditions of myasthenia continued to improve, and the child's general condition was good.

## 3 Discussion

PM is one of the rare phenotypes of cGVHD after stem cell transplantation, which accounts for about 3.4–7.7% of the incidence of cGVHD.^[12]^ To date, approx. 40 cases of PM have been reported worldwide. PM is rare in children. Thus far, no effective method of treatment has been developed.^[13,14]^ The Bohan and Peter criteria are widely applied for the diagnosis of PM.^[15]^ Most patients present with different degrees of symmetrical proximal muscle weakness that last from several weeks to several months. In some cases, dysphagia or respiratory muscle involvement also occurs. About 30% of patients develop myalgia.^[16]^ Laboratory examinations usually show increased CK levels, while alterations (usually increase) in other enzymes may include aldolase, myoglobin, LDH, AST, and ALT. The typical EMG manifestations include increased insertion potentials and spontaneous potentials, accompanied by fibrillation potentials and sharp positive waves. Pseudorigidity or complex repetitive discharges are rare. Multidirectional motor unit action potentials (MUAPs) with short duration and low amplitude are also visible in some patients, and early recruiting MUAPs.^[17]^ Pathological examination of muscle biopsy typically shows muscle fiber degeneration, necrosis, regeneration, or inflammatory cell infiltration.^[9,10]^

With the advancements in related research, myositis-specific antibodies (MSAs) and muscle MRI have been increasingly used for diagnosis and evaluation of the treatment efficacy of myositis. However, most patients have no positive MSAs, while MRI examinations show abnormal high signals of T2 fat-suppression sequences of muscle tissues, suggesting muscular inflammation and edema.^[7,17]^ Muscle biopsy plays an important role in disease diagnosis; still, not all the results of pathological examination meet the diagnostic criteria for PM.^[18]^ Stevens et al^[19]^ reported that, among 1859 patients with cGvHD after allo-HSCT, 12 had PM; however, muscle biopsy examination showed positive results in only seven patients, autoantibody was detected in eight patients, and the EMG was abnormal in five patients. In the present case, we found typical skin manifestations of cGVHD. Symmetrical proximal limb muscle weakness occurred during treatment; EMG showed abnormal results, and serum CK level substantially increased. After treatment with high-dose corticosteroids, tacrolimus, ruxolitinib, and rituximab, the manifestations improved, and serum CK level returned to a normal level. The pathological results of the patient's muscle biopsy’ were atypical, and this could be associated with the previous corticosteroid treatment before biopsy.

Corticosteroids are still used for the first-line treatment of PM.^[20]^ However, not all patients respond to therapy. For patients with strong response to corticosteroid treatment, sufficient doses and long-term treatment should be performed. Premature dose reduction can easily induce disease relapse, but this can then be successfully addressed with a further increase of the doses.^[21]^ The combination of immunosuppressive agents could further improve its effectiveness. The immunosuppressive agents include mycophenolate mofetil, imatinib, low-dose methotrexate, antilymphocyte globulin, thalidomide, hydroxychloroquine, cyclophosphamide, and TNF-α inhibitor. Despite the variations across individuals, most of these immunosuppressive agents do reach optimal effectiveness.^[3,22–24]^

Rituximab has been rarely used for the treatment of PM. Chinello et al^[25]^ reported the first case of a child with PM receiving high-dose methylprednisolone treatment in combination with rituximab. Their results showed rituximab to be highly effective in the treatment of PM. The mechanisms underlying the treatment effects could be associated with the immunosuppression mediated by the blockage of autoantibody of B lymphocytes.^[26]^ For our patients, rituximab was used for the second round of treatment and it showed good effects. However, treatment was often discontinued due to financial issues. The JAK kinase inhibitor ruxolitinib has definite effectiveness for refractory and recurrent cGVHD;^[27,28]^ nevertheless, the evidence for PM treatment is limited. In this case, low-dose ruxolitinib was used in the early stage of treatment, and it substantially improved the manifestations of cGVHD, including oral ulcers, skin rash, and liver dysfunction. The patient's muscle weakness also improved. These findings showed that continuous treatment by ruxolitinib could be effective in the treatment of PM. Moreover, no evident toxic effects were found.

## 4 Conclusion

cGVHD-related PM is relatively rare occurrence. Some patients’ prognoses are poor, which highlights the importance of early diagnosis and treatment. Corticosteroids in combination with immunosuppressive agents for the treatment could achieve good effects. Rituximab is certainly effective in treating this condition, but its efficacy in the treatment of PM needs to be further evaluated in future clinical practice.

## Acknowledgments

We are grateful to our patient and his family. We would also like to thank our colleagues for helpful discussion regarding this case. We thank LetPub (www.letpub.com) for its linguistic assistance during the preparation of this manuscript.

## Author contributions

**Conceptualization:** Yonghong Tan.

**Formal analysis:** Jinzong Lin.

**Investigation:** Jinzong Lin, Xiuli Hong.

**Methodology:** Yonghong Tan.

**Project administration:** Quanyi Lu.

**Resources:** Xiuli Hong.

**Writing – original draft:** Yonghong Tan.

**Writing – review & editing:** Jinzong Lin, Xiuli Hong, Jingyuan Lu, Quanyi Lu.
